# HIV-1 subtype C primary isolates exhibit high sensitivity to an anti-gp120 RNA aptamer

**DOI:** 10.1186/1742-4690-9-S2-P215

**Published:** 2012-09-13

**Authors:** HT Mufhandu, KB Alexandre, ES Gray, L Morris, M Khati

**Affiliations:** 1 Council for Scientific and Industrial Research (CSIR), Pretoria, South Africa; 2 National Institute for Communicable Diseases (NICD), Johannesburg, South Africa

## Background

Globally, HIV-1 subtype C is the most prevalent subtype, yet most antiretroviral drugs are developed against subtype B. UCLA1 RNA aptamer, which we previously showed neutralizes HIV-1 subtype C Env-pseudotyped viruses was examined for neutralization of subtype C primary isolates in PBMC and monocyte-derived macrophages (MDM). We also assessed the ability of subtype C to develop resistance to UCLA1 inhibition by propagating the isolates in increasing concentrations of the aptamer.

## Methods

UCLA1 was tested against clinical isolates in PBMC (6 isolates) and MDM (4 isolates) using a p24 antigen read-out. Three viruses were grown in the presence of increasing aptamer concentrations to select for resistance. The viruses were passaged every 7 days up to 12 weeks in CD8 depleted PBMC. The gp160 was sequenced, analyzed and compared with wildtype viruses.

## Results

UCLA1 neutralized 67% and 75% of viruses tested in PBMC and MDM, respectively. Overall, the aptamer neutralized one X4 and six R5 tropic viruses with IC_80_ values in the nanomolar range. Two viruses remained sensitive to the aptamer even in the presence of 4- and 12-fold increased UCLA1 concentrations. One isolate exhibited resistance after 12 weeks of propagation tolerating 12-fold the starting IC_70_. Fifty-eight amino acid changes and two insertions along the gp160 were observed. The changes observed within the V1/V2 and V3 loops confirmed our previous data shown by truncation and single point mutational analyses to confer resistance to UCLA1.

## Conclusion

UCLA1 was able to neutralize infection of primary isolates in PBMC and MDM without tropism restriction. The extensive amino acid sequence changes associated with UCLA1 resistance may indicate a high genetic barrier needed for resistance to UCLA1. This was also suggested by the low rate of resistance (only 1 of 3 isolates) observed in the study suggesting that UCLA1 is a potential anti-HIV-1 subtype C entry inhibitor drug.

